# School-based prevention of teacher and parental violence against children: Study protocol of a cluster-randomized controlled trial in Tanzania

**DOI:** 10.1186/s12889-024-19888-7

**Published:** 2024-08-31

**Authors:** Katharina Mattonet, Eliud Kabelege, Getrude Mkinga, Lena Kolwey, Mabula Nkuba, Faustine Bwire Masath, Katharin Hermenau, Claudia Schupp, Janina I Steinert, Tobias Hecker

**Affiliations:** 1 Department of Psychology, Bielefeld University, Bielefeld, Germany; 2 Institute for Interdisciplinary Research on Conflict & Violence (IKG), Bielefeld University, Bielefeld, 33501 Germany; 3 Department of Educational Psychology and Curriculum Studies, Dar es Salaam University College of Education (DUCE), Dar es Salaam, Tanzania; 4 Department of Educational Psychology and Curriculum Studies, Mkwawa University College of Education (MUCE), Iringa, Tanzania; 5 Clinic of Child and Adolescent Psychiatry and Psychotherapy, Protestant Hospital Bethel, University Hospital EWL, Bielefeld, Germany; 6 TUM School of Social Sciences and Technology, Technical University Munich, Munich, Germany

**Keywords:** School violence, Teacher violence, Parental violence, Family violence, Children, Public schools, Primary schools, Cluster-randomized controlled trial

## Abstract

**Background:**

Violence against children at home and at school is particularly prevalent in Africa and is associated with adverse and persistent health effects on children. The violence prevention intervention *Interaction Competencies with Children* -* for Teachers* (ICC-T) is an effective tool to reduce violence against children by fostering teachers’ non-violent communication and interaction skills. To enhance these effects, in the present study, ICC-T will be extended to parents (ICC-P) aiming to increase children’s experience of consistent behavior and application of non-violent discipline strategies between teachers and parents.

**Methods:**

To investigate the effectiveness of the school-based combined implementation of ICC-T and ICC-P, a cluster-randomized controlled trial with 16 primary schools in the urban district of Morogoro in Eastern Tanzania will be conducted. Both quantitative (structured interviews) and qualitative (focus group discussions, in-depth interviews, evaluation forms) methods will be used to investigate the effects on teachers’ and parents’ violence against children in home and school settings. The intervention implementation will be accompanied by a comprehensive process evaluation to assess the implementation quality of and participants’ engagement with ICC-T and ICC-P. Potential downstream effects of violence reduction will be investigated by assessing the children’s mental health and well-being.

**Discussion:**

The present study aims to provide evidence for the feasibility, acceptability, and effectiveness of the school-based combined implementation of ICC-T and ICC-P to reduce teacher and parental violence against children and contribute to children’s well-being in home and school settings.

**Trail registration:**

The clinical trial was registered at ClinicalTrials.gov (ClinicalTrials.gov, 2024) under the identifier NCT06369025 (Hecker, Preventing Physical and Emotional Violence by Parents and Teachers in Public Schools in Tanzania (ICC-T/ICC-P_Tanz) (PreVio), 2024) on April 17, 2024.

## Background

By ratifying the United Nations (UN) Conventions on the Rights of the Child [1], violence against children has been outlawed worldwide. Nevertheless, more than 1.7 billion children – 75% of all children worldwide – experience violence in their upbringing [2]. In school and at home, teachers and parents appear to use violence against children in order to regulate or correct misbehavior [3, 4]. These violent disciplinary measures may be physical acts of violence and include beatings with the use of hands or objects, shaking, pinching or kicking children as well as emotional acts of violence including harsh verbal reprimands as well as public humiliation [5, 6]. Several studies suggest that violence against children is associated with lower quality in the child–adult relationship, negative mental health outcomes, and behavioral and emotional problems that begin in childhood and may persist through adolescence and adulthood [7–11].

### Many children are still regularly subjected to violent discipline both at home and school

Prevalence rates of violence at home and in school are higher in poorer countries [12], with particularly high rates in Africa [4, 13, 14]. With violence prevalence rates of more than 89% at home and in school in East and North African countries [2, 6], prevalence rates in Tanzania [3, 14] are representative for these countries. Specifically, about 75% of children in Tanzania report having been slapped, kicked, beaten up, pushed or threatened with a weapon by relatives, parents, or guardians during their childhood [4, 15, 16]. This high rate of violence against children by parents or guardians in Tanzania reflects the current legal situation in this country, with laws permitting the use of corporal punishment in all setting as justifiable means of correcting children’s behavior [8, 17].

In school settings, violence is still legally accepted in 64 countries worldwide, mostly comprising Asian and African countries with middle to low income [18]. Systematic reviews indicate that in low- and middle-income countries, 70% to 100% of children experience physical violence by teachers, particularly in Africa [6, 19]. These reviews also reveal that physical violence in schools even remains high in countries where corporal punishment has been banned by law. Similar to the reported rates for physical violence, the prevalence rates for emotional violence perpetrated by teachers against children are reported to be high in countries across the world [20–22]. In Tanzania, 99% of teachers of secondary schools reported that they have used emotional violence against a student at least once in the past year [16] and more than 95% of primary school teachers had used at least one or more forms of emotional violence in the past one month [23].

### Teachers and parents justify physical and emotional violent discipline to maintain children’s discipline

Parents’ or teachers’ use of violence against children is influenced by various interacting factors including structural, institutional, community, interpersonal, and individual factors [24]. Community social norms and culture appear to promote parents’ and teachers’ use of physical and emotional violence to correct or shape children’s behaviors [13]. Specifically, both parents and teachers reportedly share the belief that the use of physical and emotional violence is a way to demonstrate dominance over children and thus gain their respect for educational intentions [4, 23]. Moreover, the use of violence by parents and teachers has been associated with a lack of knowledge of non-violent alternatives to dealing with children’s misbehavior or good parenting skills [4, 25], personal experiences with violence in their upbringing [23], and high-stress working conditions and economic strain [26, 27].

### Effects of violence on child development

Evidence from clinical and developmental psychology shows that violence experienced in childhood is associated with toxic stress [28]. It is well established that stress during childhood affects the developing brain and establishes permanent alterations in psychosocial and physical health. These alterations can undermine learning and affect children’s behavioral, social, and emotional functioning. Several studies suggest that violence against children is associated with lower quality in child–adult relationships, negative mental health outcomes, and behavioral and emotional problems that begin in childhood and may persist into adolescence and adulthood [7–11]. Violence against children has also been associated with impaired cognitive functioning [8, 29, 30]. Studies on children’s and adolescents’ victimization focusing on family settings and studies focusing on school settings find comparable negative effects of physical violence including physical injury and even death, poor academic outcomes, mental health, and behavioral problems [6, 19, 31–34]. Thus, violent discipline as an educational measure is counterproductive to the children’s upbringing parents’ and teachers’ beliefs about positively shaping children’s behavior. In fact, it physically, emotionally and socially harms children.

### Violence reducing interventions targeting parents and teachers

The strikingly high prevalence of violence against children calls for concentrated efforts to reduce and stop violence at different levels including: individual, family, institutional, and societal levels at large. Researchers suggest legislative reforms to prohibit and sanction the use of violent discipline in the family and at school [35]. In line with this, the UN Sustainable Development Goals 2030 initiatives including goal 16:2 [36] and the African Charter on the Rights and Welfare of the Child [37] are likely to have impacts on political changes and legal reforms in African countries and other parts of the world. In parallel, programs that promote awareness about the various detrimental effects of child violence, but also strengthen reporting and referral structures for victimized children, and provide positive parenting skills emphasizing non-violent discipline strategies to parents, caregivers, and educators’ practitioners are needed.

Regardless of global and continental initiatives, however, there is a pressing need for preventative interventions targeting the family and school level to eliminate violence against children, particularly in African countries where violence against children is rampant [18]. To this end, teachers and parents need to be made aware of the adverse and lasting effects of their use of violence against children, learn and use non-violent discipline strategies in their daily routines, and change their personal beliefs. Interventions addressing the perpetrators’ beliefs and behaviors are thus urgently needed to keep a child safe at home and at school.

Only two programs that equip teachers with non-violent action alternatives have been implemented and rigorously evaluated in Africa: the Interaction Competencies with Children – for Teachers (ICC-T) [3, 5, 38, 39] and the Good Schools Toolkit [40]. The latter program focuses on extensive staff training and coaching on non-violent disciplinary methods and involves classroom activities for students. In follow-up evaluations, students assigned to the intervention schools reported a significant decrease in the frequency of past-week physical violence use by teachers, compared to students in control schools [40]. The ICC-T approach is particularly feasible in low-resource contexts as the costs for the implementation are comparably low. The core component of the program is a manualized one-week training workshop focusing on the basic competencies in the work with children, including non-violent action alternatives. ICC-T is very flexible and participatory as it engages the participants in tailoring the program according to their knowledge and needs. This makes ICC-T successful with people from various countries and cultural or educational backgrounds. The ICC-T program is designed in such a way that local professionals with a background in social work, psychology or the like are also able to implement the program with teachers [41]. In cluster-randomized control trails (CRCTs) in Tanzania and Uganda, it was shown that teachers who participated in ICC-T applied significantly less violence against children compared to teachers in the control group. This was not only reported by the teachers themselves, but also by the students who were blinded with regards to the treatment allocation of their school [3, 42].

However, children do not only experience violence at school but also in their home. In order to effectively protect children from violence in their upbringing, preventive work with those who most frequently and intensively use violence against children, i.e. parents and teachers, is of central importance. A number of previous studies have evaluated the benefits of parenting programs that emphasize the use of non-violent discipline techniques [43, 44]. One example is the Parenting for Lifelong Health (PLH) [45] program which was developed, delivered, and tested mainly in South Africa [46], but with adapted versions also in other African countries including Tanzania [47] and Kenya [48]. The program focuses on preventing child maltreatment and involvement in other forms of violence at the family level. The experimental evaluation of the program pointed to improvements in the parent–child relationships, child attachment, and of the cognitive and socio-emotional development of children as well as to reductions in the family mental health distress [46]. The Irie Homes Toolbox is another parenting program for families in low- and middle-income countries [49], with studies documenting increases in parents’ friendly interaction with their children and decreases in the use of harsh punishment among parents who have received the intervention [50, 51]. These interventions focus on parenting skills. Yet, interventions focusing on stopping or reducing violent discipline at home and school by equipping parents and teachers with non-violent discipline strategies and addressing changes in personal beliefs and questioning social norms are also highly needed to keep a child safe at home and school.

### Aims and Objectives—children experience consistency in the behavior and application of non-violent discipline strategies between teachers and parents

Following the socio-legal research’s approach to begin at school-level to reduce violence against children [35], a school-based approach including both teachers and parents has three key advantages: a) Children experience consistency in the behavior and application of non-violent discipline strategies between teachers and parents, b) parents of different social, economic, and educational backgrounds can be motivated to participate in the intervention, and c) using the existing infrastructure of schools reduces costs and improves the reach and scalability of the program. In line with this, researchers also argue that parents and teachers learn from one other [3].

This research project follows up on extensive evidence on the feasibility and effectiveness of ICC-T [52] and on evidence from one study of ICC-P which was conducted in an uncontrolled design with parents [53]. By combining ICC-T and ICC-P in a school-based intervention, the present study uses a unique and novel intervention approach as it will focus on reducing violence at family and school level at the same time. The intervention approach will enhance teachers’ and parents’ interaction competencies with children. This study is expected to improve the holistic environment for children at school and at home. We hypothesize that the school-based preventive intervention will significantly reduce the use of parents’ and teachers’ physical and emotional violence against children and will have a positive impact on children’s well-being and mental health.

## Methods

### Design

**Fig. 1:**
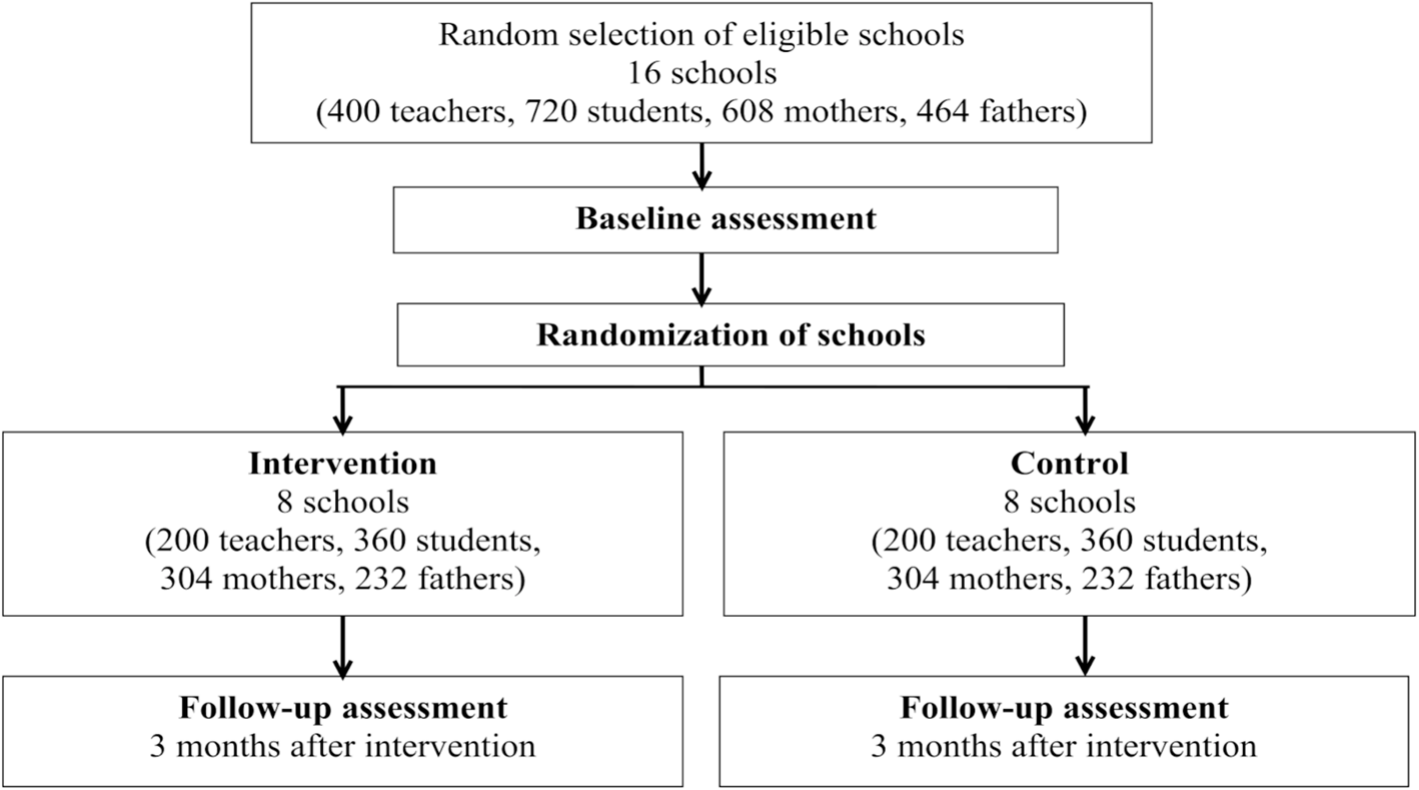
Flow chart of the study design

We will use a two-armed school-based CRCT including 16 primary schools in the region of Morogoro, Tanzania. Eight of 16 selected schools will be randomly allocated to the intervention group, which will receive the school-based training (ICC-T for teachers, ICC-P for parents), and the other eight schools will be allocated to the control group without any intervention. The study will include two data collection phases, one baseline assessment before the intervention allocation and one follow-up assessment approximately three months after the allocation and delivery of the intervention (c.f., study flow chart in Fig. 1 & time-line in Table 1).

**Table 1:** Study time-line

	Study period		
	Baseline (t_0_)	Intervention (period: 6 weeks)	Follow-up (3 months after intervention; t_1_)
Allocation (after baseline)	x		
ICC-T & ICC-P training		x	
Primary outcome: violence by teachers & parents	x		x
Secondary outcomes (i.a.; stress)	x		x
Process evaluation		x	
Focus group discussions		x	
In-depth interviews			x

### Study setting and sampling

This intervention study will be implemented in public primary schools in Morogoro in Eastern Tanzania, particularly in the urban district of Morogoro. Based on geographical locations of districts in the Morogoro region, the Morogoro urban district council was purposely selected for the study for feasibility reasons. For randomization, we used random.org [54]. First, we created a list of all eligible schools (38) fulfilling our inclusion and exclusion criteria from all wards (smallest administrative unit) located within the urban district of Morogoro. Second, based on our a priori power analysis, we randomly selected 16 wards and one school in each ward. We selected one school from each ward to avoid cross-over effects between the study conditions due to proximity of schools. Third, pairs of schools were formed due to primarily logistical reasons: Due to the short funding period (12 months) of this research project, the intervention will be delivered school by school. Once the first pair of schools has completed the baseline assessment, the random allocation assigns one school to the intervention and the other to the control condition and the implementation of the intervention starts directly after allocation. The allocation of the schools will be performed by the research team witnessed by two teachers from each school.

#### Schools

To be eligible for inclusion in the study, the schools have to meet the following criteria: The school is a public primary day school within the Morogoro urban district and is a mixed-gender and uniform language (Swahili) school. Public primary schools are state owned school. Thus, they are guided by Tanzania education policy including free education and the guideline for violent discipline. The school has at least 45 students in the selected grade. However, if the selected school has less than 45 students in the selected grade, then it will be combined with a nearby selected public primary school which must be within 15 km (km) from the respective school with too small class size. An equal number from each school cluster will be selected to be invited to participate in the study. The school must have at least 25 but no more than 50 employed teachers. The upper limit of 50 teachers is due to difficulties to providing the intervention. In case of less than 25 teachers, the selected school will form a school cluster together with another near-by (i.e., within 15 km) public primary school, with all teachers of both schools will be included in the study.

#### Participants

In this study, students, their parents, and teachers will be assessed at baseline and follow-up stage. The research team will cooperate with the district educational office to receive the name and ward locations (administrative division of a district) of the schools. To recruit teachers, the research team will collaborate with the head of school from the selected school and invite all teachers employed at the school. In cooperation with the collaborating teacher in the selected school, the research team will select 45 students from fifth school grade to participate in the study. Parents of the selected students will also be invited to participate in the study. The research team in collaboration with the school management will prepare letters for the parents of selected students to participate in the study. The sealed letters will be given to the student to send to his or her parents. In addition, the school management will send a text message or call the parents to consider the invitation.

Inclusion criteria for students are being enrolled in fifth school grade of a public primary school within the selected schools. Further students’ inclusion criteria are being of age between 9 and 13 years and living with their caregiver or parents. Exclusion criteria for students are severe mental disabilities that make it impossible to follow the interview questions. Inclusion criteria for teachers is to be employed at an eligible school. Exclusion criteria for teachers are acute intoxication of alcohol, drugs, or any other substance which makes it impossible to follow the interview questions. Inclusion criteria for parents are that their child is participating in the study and is enrolled in a study’s eligible school. Being a parent is understood as being the social parent currently taking care of the child independent of the biological relationship to the children. Thus, a parent can be biological parents, step-parents, foster parents, relatives, etc. The parents have to live with the child and in reachable distance to the child’s school. Exclusion criteria for parents are severe mental disabilities and acute intoxication of alcohol, drugs, or any other substance that make it impossible to follow the interview questions.

*A priori* sample size and power analyses using The Shiny CRT Calculator [55] for the CRCT with pre-post assessment was conducted [56] with a critical significant level of 5%, a power of 80%, an expected moderate effect size of ƒ = 0.25, and an assumed intra-class correlation coefficient of .05. Previous ICC-T trials in Tanzanian secondary schools revealed moderate to large effects on student-reported and teacher-reported violence by teachers, respectively [3, 4, 39, 53]. Assuming no dropout at the school level and accounting for potential dropout on the participant level of 25%, and eight clusters per arm, 25 to 28 participants within a cluster would result in sufficient power. To account for parents’ response rates that are expected to be lower than 100%, parents from 45 randomly selected students will be invited to participate. Based on an expected approximated 85% response rate of invited mothers and 65% of invited fathers, 38 mothers and 29 fathers are assumed to participate in the study. In addition, 25 teacher per school will be included in the present study. This results in a sample size of 720 students, 608 mothers, 464 fathers and 400 teachers across 16 schools. Based on these assumptions, an *a priori *power of ß = .94 for students, for mothers ß = .90, for fathers ß = .82, and ß = .76 for teachers was calculated.

### Procedures

Before data collection, research assistants will be selected by the research team and trained on data collection procedures in a five-day-workshop followed by a one-day-pilot run. Research assistants will be recruited based on the following characteristics: holding at least a bachelor degree from university and being fluent in English and Swahili language. Having prior experience in research projects on social, education, and/or health related matters is an additional advantage. All research assistants will remain blind as to whether the school was assigned to the control or intervention group.

Before data collection, informed consent of participants will be collected. The research team will explain the objectives of the study to the selected students and will provide them sealed letters of invitations for both parents to come to school. Upon parents’ arrival to school, the research team will explain the objectives of the study, and provide them with the study information sheets and the informed consent forms. Parents must read the study information themselves or with help of the research team if they are not able to read and write. Parents will then sign a consent form holding information on the aims of the study, confidentiality of the data, and the rights to withdraw from the study at any point in time during the progress of the study and without any negative consequences for participation. In addition, parents will have to provide written consent to allow their children to participate in the study. With parent’s permission and prior to the interview, the research team will inform the child orally and will ask the student if he or she is willing to participate in the study. Teachers will individually be invited to a discrete and quiet place within the school premises and be given detailed written and oral information on the aims of the study, confidentiality of their data, and their rights to withdraw from the study at any point in time during the progress of the study and without any negative consequences. Teachers will sign the consent form prior to starting the interview.

For data collection, baseline and follow-up assessments will include structured interviews for students, parents, and teachers. Participants will be interviewed by trained interviewers in the local language (Swahili). In the interview, the interviewer will directly enter participants’ responses into an Android tablet using the survey tool OfflineSurveys Pro version 1.81, based on Limesurvey version 3.27.30. The interview holds standardized introductions and administration procedures to ensure high objectivity and reliability during the data assessment. To ensure participants’ full understanding, all interviews and communications will be conducted in Swahili language. Swahili versions of the instruments that have been translated by independent translators following the scientific guidelines [57] will be used. At the end of each interview parents will be compensated (five thousand Tanzanian shillings) for their transport.

The assessment procedure will be repeated in the same way approximately three months (12 weeks) after the complete delivery of the intervention. Given the nature of the interventions, all participants will be masked during baseline assessment, however, participants will be unmasked at follow-up assessments. Nevertheless, assessors will be blind throughout the study as the interventions only target parents and teachers.

### Intervention

In this study, the interventions will consist of two mutually exclusive conditions for the teachers or parents: a) ICC-T – the intervention for teachers which will consists of a five-day-training (8 h per day) or b) ICC-P – the intervention for parents which will consist of a three-weekend-day consecutive training session (8 h per day) and a fourth day as a refresher day six weeks later. The ICC-T and ICC-P training concepts are based on the childcare guidelines of the American Academy of Pediatrics [58] and Dreikurs et al. [59]. The ICC program offers participants (parents & teachers) a basic training in essential interaction competencies with children. The intervention focuses on non-violent interaction strategies and on encouraging warm, sensitive, and reliable adult–child relationships [41]. ICC-P is an adapted version of ICC-T, shifting from school to family context [53] with the aim to achieve the greatest possible agreement in content between ICC-T and ICC-P. It has been successfully field-tested [53].

ICC-T [3, 38, 39, 42] and ICC-P [53] aim to prevent harsh and violent discipline in school and home settings respectively by improving the relationship between children and teachers as well as children and parents, by confronting and altering the teachers’ and parents’ favorable attitudes towards violence and enabling them to use non-violent discipline strategies. ICC-T and ICC-P follow four key principles which guide the implementation: 1. Participative approach: teachers and parents are encouraged to contribute actively in the training; 2. Confidential workshop atmosphere: participants can freely reflect and share their daily challenges, needs, and personal experiences in dealing with children’s behavior; 3. Intensive practice combined with theoretical inputs: parents and teachers practice to integrate the learned skills into their daily interactions; 4. Training sustainability with various activities including repetition of the content, self-reflection of personal experience, team support and peer support groups, networking and ongoing supervision. These key principles guide the ICC-T and ICC-P training workshops that focus on five core components (c.f., Table 2).

**Table 2:** Descriptions on intervention modules

Brief name	1. Interaction Competencies with Children – for Teachers (ICC-T) 2. Interaction Competencies with Children – for Parents (ICC-P)
Rationale, theory, & goals	Maltreatment prevention and improvement of adult–child relationship; based on attachment, behavioral, and social learning theories and the guidelines set forth by the American Academy of Pediatrics [58]; violence prevention components were grounded in the work of Dreikurs et al. [59]
Materials	The ICC-T manual^a^ with the respective materials (including facilitator instructions, handouts, theoretical inputs, instructions for discussions, & role-plays) is freely available. The ICC-P manual is currently under development and can be requested by the authors
Procedure	1. ICC-T begins with a welcome session in which the expectations, wishes, and concerns of the trainees are explored. Five core components form the content of ICC-T: teacher-student interaction (3 sessions at 90 min), maltreatment prevention (5 sessions at 90 min), effective discipline strategies (8 sessions at 90 min), supporting burdened students (2 sessions at 90 min), and implementation (2 sessions at 90 min). Participants form peer support groups and are invited to seek advice from trainers virtually if needed. At the beginning and the end of the training, teachers are asked to evaluate the feasibility of the intervention. A participation of four entire days leads to certification of attendance 2. ICC-P begins with a welcome session in which the expectations, wishes, and concerns of the trainees are explored. Four core components form the content of ICC-P: adult–child interaction (3 sessions at 60–90 min), maltreatment prevention (4 sessions at 60–90 min), effective discipline strategies (7 sessions at 60–90 min), and implementation (1 sessions at 90 min). A refresher day after 6 weeks enables practice, repetition, and exchange with peers and trainers. The participants form peer support groups and are invited to seek advice from trainers virtually if needed. The first two days are conducted in same gender groups, the third day and the refresher days in mixed gender groups. At the beginning and the end of the training, parents are asked to evaluate the feasibility of the intervention. A participation of 3 out of 4 days (including the refresher day) leads to certification of attendance
Provider	Two trainers with background in psychology and/or teaching per school
Location	At the premises of the selected schools
Duration	1. ICC-T: 5 days (8.5 h per day) 2. ICC-P 3 days + 1 refresher day (8 h per day)
Tailoring	Tailoring is one of the key principles of ICC intervention modules: Trainees are invited to actively participate, tailor the program, and develop their own strategies for implementing the training content in their daily work with flexibility and fidelity
Modifications	There are no modifications of the intervention
Fidelity	To increase fidelity comprehensive materials are provided. Trainers apply all the required materials

*Note.* The described intervention modules have been adapted from the Template for Intervention Description and Replication (TIDieR) checklist [60]. For detailed intervention descriptions: c.f., Kirika & Hecker [41]; Kabelege et al. [53]

^a^The *ICC-T* training manual is available at [61]

#### Intervention procedures

Trained facilitators with well-defined background in psychology and adult-teaching will implement the ICC-T and ICC-P interventions within the premises of the selected schools. Eligible research assistants will work closely with trainers throughout the intervention. Participation in ICC-T and ICC-P respectively will be free of charge for all participants. Participants will be provided with food (breakfast & lunch) and beverages during training. Information explaining in detail the training procedure and topics will be given to each participant.

The training of parents will take place over three consecutive Saturdays and one refresher day delivered after six weeks. ICC-P will be implemented for both female and male parents in groups of 20 to 25 participants. The parents will be individually invited for training and will have gender-separate workshops on the first two workshop days with the intention to maximize participation of female and male parents [62] and to encourage parents to freely express their beliefs about discipline measures used with their children. On the third and fourth day, female and male parents will participate jointly in one training group as they will be trained on alternative discipline and good parenting skills. Teachers from the selected schools will be trained in ICC-T in groups of approximately 25 participants on five consecutive days in a week during school holidays in 2024. All trainings will take place within school premises.

To monitor the fidelity, acceptability, and feasibility of the training, a process evaluation will be conducted during the training sessions of ICC-T and ICC-P. The process evaluation will utilize standardized evaluation forms completed by trained research manager observing the program sessions (rating participants’ engagement & facilitators’ performance) and standardized reflection forms completed by facilitators after each session (rating their satisfaction with the session and specific activities covered). Furthermore, for each training day, five randomly selected participants will be asked to fill out a purpose-built questionnaire on their perceived comprehension of that day’s training content and the method used by the facilitator for delivering the content during that day. At the end of the workshop, all participants will evaluate the session content, the methods used, and the performance of trainers using a standardized questionnaire.

The participants’ experiences with and thoughts on the intervention will be assessed in one focus group discussion (FGD) with 10 parents after the refresher day and one FDG with 10 teachers two weeks after the implementation and one FGD with all facilitators after the completion of all intervention workshops. Ten in-depth interviews with five teachers or parents who have reported decreases in violence perpetration post-intervention and five teachers or parents who have reported no changes (or even increases) in violence perpetration. The FDGs and in-depth interviews will be audio recorded.

#### Control

There will be no training delivered to participants from control schools. Data will be assessed at baseline and follow-up with the same procedure in control and intervention schools. During the present study, the control schools will not be part of any other, similar interventions.

### Outcomes

Quantitative data will be collected to investigate the effects of ICC-T and ICC-P on the teachers’ and parents’ use of violence against children in home and school. As primary quantitative outcome, violence by teachers in school and by parents at home will be assessed. The secondary quantitative outcome measures include parents’ and teachers’ favorable attitudes towards violence, children’s mental health problems, children’s quality of life, peer violence experienced by children, parent–child relationship and teacher–child relationship, perceived stress of all participants, school climate experienced by teachers, teachers’ self-efficacy, teachers’ decision making, teachers’ and parents’ normative beliefs about violence, and intimate partner violence experienced by female parents. All primary and secondary quantitative outcomes will be collected at baseline and follow-up data assessment. In addition, socio-demographic data on participants’ age, gender, and living situation will be assessed. Apart from this, children will be asked to identify the two most caring persons for them personally and parents and teachers will be asked on their household earnings.

Qualitative information will be collected to complement the quantitative data with FGDs and in-depth interviews for process evaluation and qualitative end-line evaluation. A process evaluation will be conducted to assess the quality and fidelity of the implementation of the intervention, participants’ engagement with the intervention, and whether the intervention achieved its goals in terms of its primary and secondary quantitative outcomes. The FDGs will assess the parents’ and teachers’ experiences with and thoughts on the intervention. The in-depth interview will assess parents’ and teachers’ perceived improvement of violence reduction. For qualitative information on process evaluation, standardized evaluation as well as reflection forms will assess the staff members’ experiences with conducting the intervention.

#### Primary quantitative outcomes

##### Parental violence

The use of emotional and physical violence by parents against children at home will be measured with adapted versions of the Conflict Tactics Scale (CTS) [3, 63, 64], on the one hand by children’s self-reported violence experiences and on the other hand by parents’ self-report on the use of violence. For the present study, the CTS will include 9 items on experienced physical violence, 7 items on experienced emotional violence, and 5 items on emotional maltreatment. The items will be answered on a 7-point Likert scale ranging from 0 (this has never happened in the past week) to 6 (not past week, but before). CTS-scores on emotional and physical violence will be derived by summing up the respective item scores, whereby higher scores indicate higher levels of parental violence. A previous study in Tanzania assessed parental violence with the adapted CTS versions and revealed good psychometric properties [53].

In addition, during the quantitative interview 4 CTS-items will be body mapped by asking the children where on their body they experienced the emotional (shout & call dumps) and physical (hit & slap) violent act by parents. The affected body parts will be documented on a sheet of paper with a body-icon. Promising results for body mapping have been shown for trauma research in the context of refugees [65].

##### Teachers’ violence

An adapted version of the CTS [3, 63, 64] will be used to assess the emotional and physical violence by teachers at school, captured through both children’s and teachers’ self-reports. The CTS will include 9 items on physical violence, 7 items on emotional violence. For children’s report, 2 additional purpose-built items on witnessed violence by teachers will be assessed. The teacher version uses the same answer scale and scoring as the CTS versions for parental violence (c.f., for more details outcome description about parental violence). Previous studies in East Africa assessing teachers’ violence revealed acceptable psychometric properties [14, 66, 67].

In addition, during the quantitative interview 4 CTS-items will be body mapped by asking the children where on their body they experienced the emotional (shout & threaten) and physical (hit & pinch) violent act by teachers. The affected body parts will be documented on a sheet of paper with a body-icon. Promising results for body mapping have been shown for trauma research in the context of refugees [65].

#### Secondary quantitative outcomes

##### Attitudes towards violence

Favorable attitudes towards physical and emotional violence will be assessed from parents and teachers with an adapted version of the CTS [3, 63, 64]. Sixteen items are formulated as a statement beginning with “When children/students do something wrong, I think it is OK to …”. Each item ends with the respective act of physical or emotional violence. The items are answered on a 4-point Likert scale ranging from 0 (never OK) to 3 (always or almost always OK). Subscale items are then sum scored for each parent or teacher with higher values indicating parent’s or teacher’s more favorable attitudes towards physical or emotional violence. The adapted CTS was used before to assess teachers’ self-reported attitudes towards violent discipline in Tanzania [3, 23].

##### Children’s mental health problems

The children’s emotional and behavioral problems will be assessed with the short version of the Pediatric Symptom Checklist – Youth Report (PSC-17-Y) [68, 69]. PSC-17-Y includes 17 items rated on a 3-point Likert scale ranging from 0 (never true) to 2 (often true). These items will be summed up to a total score of emotional and behavioral problems ranging from 0 to 34. The PSC-17-Y will be answered by the children and parents [68]. Good psychometric properties have been shown in adapted PSC-versions in a sample of HIV-infected children in Botswana [70] as well as for school children in Uganda [71].

##### Children’s quality of life

The KIDSCREEN-10 Index will assess children’s global quality of life conceptualized as a multidimensional construct covering physical, emotional, social, and behavioral aspects of well-being and functioning [72–74]. Ten items referring to the past week on a 5-point Likert scale ranging from 0 (not at all) to 4 (extremely) will be answered by both the children and parents. An additional item will ask about general health which can be rated as ‘poor’, ‘fair’, ‘good’, ‘very good’, and ‘excellent’. Negatively worded items will be recoded, so that higher scores for all items indicate higher levels of children’s global quality of life. All items will then be sum scored capturing children’s global quality of life. KIDSCREEN-10 has been used in clinical and epidemiological studies in Europe, North and South America, Africa, and Asia, revealing cross-cultural validity [72].

##### Peer violence

The 24-item version of the Multidimensional Peer Victimization Scale (MPVS) [75] will assess children’s experiences of violence by peers using six subscales: physical victimization, verbal victimization, social manipulation, attacks on property, electronic victimization, and social rebuff. Four items for each category referring to the past week will be answered on a 3-point Likert scale ranging from 0 (not at all) to 2 (more than once). The total victimization score will range from 0 to 32 with subscale scores ranging from 0 to 8, whereby higher scores indicate that a child has experienced more peer victimization. Good psychometric properties have been shown for the original 16-item and the 24-item version of the MPVS [75].

##### Adult–child relationship quality

The People In My Life (PIML) [76, 77] will be used to assess the quality of the parent- as well as teacher–child relationship. Nineteen PIML Parent Factor items will assess quality of children’s self-reported relationship with their parents. The Parent Factor items will be assessed separately for the social mother and father. For teacher–child relationship, 15 items of the Teacher/School Factor items will assess the children’s self-reported relationship with their teachers. All items will be answered on a 4-point Likert scale ranging from 0 (Almost never or never true) to 3 (Almost always or always true). Negatively worded items will be recoded, so that higher scores for all items indicate higher levels of children’s global relationship quality. The Parent Factor items are sum scored as indicator of children’s global quality of their relationship to parents. The Teacher Factor items are sum scored as indicator of children’s global quality of the relationship to their teacher. The psychometric properties of the PIML have been shown to be good [77, 78].

In addition, the parents’ relationship to the own child will be assessed by the Child-Parent-Relationship Scale-Short-Form [79]. Parents will answer 15 items on a 5-point Likert scale ranging from 1 (definitely does not apply) to 5 (definitely applies). Validation studies done in different contexts [80, 81] confirmed the validity of the scale.

##### Perceived stress

The children’s self-reported perceived stress will be assessed with the Perceived Stress Scale for Kids [82] consisting of 11 items referring to the past week that are answered on a 5-point Likert scale ranging from 0 (never) to 4 (always). Negatively worded items will be recoded. The items are sum scored. Higher scores indicate higher levels of stress. Studies on the reliability and validity of the scale showed promising results [82].

Parental self-reported perceived stress will be assessed through an adapted version of the Parental Stress Scale [83]. Parents will answer 18 items about the past week on a 5-point Likert scale ranging from 1 (strongly disagree) to 5 (strongly agree). Negatively worded items will be recoded. The items are sum scored. Higher scores indicate higher levels of stress. The scale has been validated in different samples [84, 85].

Teachers’ self-reported perceived stress will be assessed through the Teachers Stress Inventory [86]. 19 items referring to the past week on a 5-point Likert scale ranging from 0 (never) to 4 (always) will be answered by teachers. Negatively worded items will be recoded. The items are sum scored. Higher scores indicate higher levels of stress. Psychometric properties have already been validated in the context of South Africa [87].

##### School climate quality & teachers’ decision making

The School Level Environment Questionnaire [88] will be used to assess school climate and teachers’ decision making based on teacher report. The Affiliation Subscale will assess school climate. Therefore, 7 items will be answered on a 5-point Likert scale ranging from 0 (strongly disagree) to 4 (strongly agree). Negatively worded items will be recoded. The items are sum scored. Higher scores indicate higher quality of school climate. The Participatory Decision Making Subscale [89] will assess teachers’ decision making strategies. Teachers will answer 7 items on a 5-point Likert scale ranging from 0 (strongly disagree) to 4 (strongly agree). Negatively worded items will be recoded. The items are sum scored. Higher scores indicate better decision-making strategies. Promising psychometric results have been shown in the context of the southwestern United States [89] and also with an adapted version in the context of South Africa [90].

##### Teachers’ self-efficacy

The Teachers Sense of Efficacy Scale [91] will assess the self-efficacy reported by teachers. Twelve items asking about how much they as teachers can do in specific situations will be answered on a 5-point Likert scale ranging from 0 (nothing) to 4 (a great deal). The items are sum scored. Higher scores indicate higher levels of self-efficacy. The Teachers Sense of Efficacy Scale has been used in the context of South Africa [92] and has shown to have good psychometric properties [93].

##### Normative beliefs about violence

Normative beliefs about violence are assessed using purpose built items answered by parents and teachers with 4 items. Two items refer to witnessing violence in the past week at school/in the community using a 6-point Likert scale ranging from 0 (never) to 5 (more than 10 times) and two items refer to general thoughts about disciplining children using a 4-point Likert scale ranging from 0 (never OK) to 3 (always or almost always OK). The respective two items are sum scored. Higher scores indicate stronger normative beliefs about violence.

##### Intimate partner violence

Intimate partner emotional, physical and sexual violence will be assessed for female social parents using purpose-built items from a combination of studies [94, 95] and the CTS. Eight items will be answered by ‘yes’ (1) or ‘no’ (0). If ‘yes’ is indicated, follow-up questions on the time frame and frequency ranging from 1 (once) to 3 (many) will be asked. The items are sum scored. A higher score indicates a higher level of intimate partner violence.

#### Process evaluation and qualitative end-line evaluation

##### Qualitative information on the quality and fidelity of the intervention

The quality of the intervention will be assessed through participants’ and facilitators’ assessments of individual sessions and their components and of the perceived facilitator performance, captured through standardized assessment forms as well as FGDs and in-depth interviews. Fidelity and facilitator performance will additionally be assessed through staff observers who will capture whether any session content remained uncovered or was poorly delivered.

##### Qualitative and quantitative information on participants’ engagement with the intervention

Participants’ attendance of each program session and individual session components will be captured based on detailed attendance records. In addition, participants’ engagement in the session will be assessed through standardized assessment forms filled out by participants themselves, facilitators and staff observers. These will assess which components of the intervention were particularly well liked among participants and which ones were perceived as rather challenging/ineffective. Lastly, participants’ engagement with the intervention will further be discussed in FGDs and in-depth interviews.

##### Qualitative information on functioning of intervention and violence reduction

The FGDs and in-depth interview will assess the parents’ and teachers’ perceived improvements in their use of alternative discipline strategies and the potential reduction in violence against children after their participation in the intervention. The FGDs and in-depth interviews will also be set up to elicit possible mechanisms and barriers of change.

#### Measures against *bias*

The risk of bias will be minimized and the validity of the findings will be increased through several methodological strategies. First, randomly sampling schools will counteract selection bias. Second, we will use previously validated and contextually appropriate instruments and thorough training of interviewers to counteract potential reporting biases and validity threats. Third, persons involved in collecting data will remain blind to the treatment conditions of the schools as the allocation to intervention and control group will be executed at the cluster level by research team members not collecting data. Fourth, carrying out analyses of data on the level of groups as randomized (intent-to-treat) will avoid incomplete accounting of participants and outcome events.

#### Ethical considerations

To protect participants’ identity, pseudonymization of collected research data will be ensured by creating a random participant code and the use of separate databases. The assignment of data from different assessment points, which is necessary due to the longitudinal study design, will be completed using the participant codes. All content collected or discussed during the investigation will be kept strictly confidential. After the completion of data collection and data quality checks, data will be fully anonymized.

As behavioral intervention studies are minimum risk studies, adverse events as a consequence of the intervention itself are not expected for our study. However, participants will be asked about personal experiences with child maltreatment, the use of or exposure to violence, their stress, and problematic family relations. This information is of intimate nature and may be perceived as stigmatizing. Participants will be protected by keeping all information confidential. Due to the interview’s content on violence, participants may be placed under mental strain depending on the burden of traumatic or aversive events and mental symptoms. High stress can lead to negative memories, emotions and physical reactions, concentration difficulties, and feelings of fatigue. In addition, it can be assumed that participants with traumatic and aversive experiences and mental symptoms are no less confronted with negative thoughts, feelings and memories in their daily experience than during the interview. Participants will be informed about the content of questions prior to the interview. Awareness about the possible occurrence of unpleasant feelings and emotions will be raised. In case participants experience severe psychological stress during the interview, short-term psychological support will be offered immediately.

## Data analyses

### Quantitative outcomes

To statistically investigate the study’s main research question whether ICC-T and ICC-P have a decreasing effect on teachers’ and parents’ use of violence against children in home and school settings, interaction effects of time*intervention will be modeled on the primary outcomes of teachers’ and parents’ violence use against children reported by teachers, parents and children respectively in mixed models for repeated measures accounting for cluster effects of participants’ nested in school. Secondary outcomes will be analyzed for potential time*intervention with mixed models. In case of significantly (*p* < 0.05) zero-inflated outcomes, model approaches for non-normally distributed data (e.g., Poisson, negative binomial) will be used. In case of unstable models due to potential outliers, variables will be winsorized (quantiles 5% & 95%) for model robustness. In case of missing data at random, multiple imputation methods will be used. Models will be corrected for multiple testing. In addition to the main inferential analyses, the prevalence of maltreatment and violence in different settings as well as children’s mental health and well-being from baseline assessment data will be captured based on descriptive statistics. Results will be presented including appropriate effects sizes and with a measure of precision (95% confidence intervals). Effect size of η^2^ ≥ 0.01, η^2^ ≥ 0.06, η^2^ ≥ 0.14 will be considered representing a small, moderate, and large effect, respectively [96].

#### Qualitative information

FGDs and in-depth interviews will be recorded, transcribed verbatim, and translated to English. Transcripts will be analyzed by two team members (JS & CS) using qualitative content analysis in Atlas.ti.

## Discussion

Several studies suggest that violence against children is associated with lower quality in the child–adult relationship, negative mental health outcomes, and behavioral and emotional problems that begin in childhood and are very likely to persist throughout adolescence and adulthood [7–11, 32]. Studies show that nine out of ten countries with the highest rates of violent discipline use against children are in Africa [2, 6].

Despite attempts to legally banning violence against children in various African countries, in Tanzania violent discipline is still permitted [97–99]. Preventive interventions are promising to reduce violence against children by directly targeting the structural engines of school and family. In particular, the ICC program offers parents and teachers a basic training in the essential interaction competencies in interacting with children [41]. The intervention focuses on non-violent interaction strategies and on encouraging warm, sensitive, and reliable adult–child relationships. ICC enhances awareness for violence and its negative consequences and reduces favorable attitudes towards as well as the use of violence against children. Thus, to prevent violence, ICC is a bottom-up approach that targets the schools and families as engine to effectively reduce violence against children [35].

In the present project, ICC-T as well as ICC-P will be delivered together within a school-based approach. With this unique approach of combining ICC-T and ICC-P, it is expected for the present research project that the intervention will especially foster the children’s consistent experience of non-violent discipline behaviors by their teachers and parents. By reducing violence against children, we aim to contribute to the decrease of children’s risk of adverse mental health outcomes that are associated with violence exposure.

To investigate the effectiveness of ICC-T and ICC-P on shared school-ground, a CRCT with 16 schools will be conducted. CRCT with pre-post intervention assessment allow for strong tests to investigate how violence use and favorable attitudes towards violence might change over a short period of time within participants that received the intervention as well as between participants comparing intervention and control condition. The use of violence assessed pre- and post-intervention will help to understand temporal changes in violence use and experience over short time. Besides these strengths of the present design, extended and multiple occasions of measurement may be helpful to understand the sustainable effectiveness of the intervention. If, as in the present study, the initial effectiveness after three months is confirmed, investigating the long term effectiveness will be highly promising. Especially well-being and health as downstream effects of violence reduction are promising to investigate in long term settings.

Sixteen schools will be selected from one region (Morogoro) in Eastern Tanzania. Conducting the present project with participants specifically from the urban districts of Morogoro, it might be possible that diverse nuances in societally influenced beliefs on violence use might differ between regions of Tanzanian. If the combination of ICC-T and ICC-P reveals effective reduction of violence use against children larger studies across various Tanzanian regions and even across various countries would be important.

The present project is aiming to reduce children’s violence exposure by educating teachers and parents on non-violent interaction competencies. Through this, the present project contributes to the efforts of Pan-African [100] and United Nations Conventions on the Rights of the Child [1] to end all violence against children.

## Data Availability

Anonymized quantitative and qualitative data will be made publicly available without expiration date in the online repository Open Science Framework (OSF) [101].

## Abbreviations

CRCTs: Cluster-randomized control trails
CTS: Conflict Tactics Scale
FGDs: Focus group discussions
ICC: Interaction Competencies with Children
ICC-T: Interaction Competencies with Children—for Teachers
ICC-P: Interaction Competencies with Children—for Parents
Km: Kilometers
MPVS: Multidimensional Peer Victimization Scale
No.: Number
OSF: Open Science Framework
PIML: People In My Life
PLH: Parenting for Lifelong Health
PSC-17-Y: Short version (17 item) of Pediatric Symptom Checklist – Youth Report
TIDieR: Template for Intervention Description and Replication
TUM: Technical University Munich
UN: United Nation
